# Opportunistic fungal infections in persons living with advanced HIV disease in Lagos, Nigeria; a 12-year retrospective study

**DOI:** 10.4314/ahs.v20i4.9

**Published:** 2020-12

**Authors:** Rita Oladele, Folasade Ogunsola, Alani Akanmu, Katie Stocking, David W Denning, Nelesh Govender

**Affiliations:** 1 University of Lagos College of Medicine, Medical Microbiology & Parasitology; 2 University of Lagos College of Medicine; 3 University Hospital of South Manchester NHS Foundation Trust; 4 The National Aspergillosis Centre, University Hospital of South Manchester, The University of Manchester, Manchester Academic Health Science Centre, Manchester, UK; 5 National Institute for Communicable Diseases

**Keywords:** Opportunistic fungal infections, ART Adherence, Advanced HIV disease

## Abstract

**Introduction:**

Nigeria has a large estimated burden of AIDS-related mycoses. We aimed to determine the proportion of patients with AIDS-related opportunistic fungal infections (OFIs) at an urban antiretroviral treatment (ART) centre in Nigeria.

**Methods:**

A retrospective analysis of a cohort of ART-naïve, HIV-infected patients, assessed for ART eligibility and ARTexperience at the PEPFAR outpatient clinic at Lagos University Teaching Hospital over a 12-year period (April 2004-February 2016) was conducted.

**Results:**

During this period, 7,034 patients visited the clinic: 4,797 (68.2%) were female; 6161 patients had a recorded baseline CD4 count, and the median CD4 count was 184 cells/µl (IQR, 84–328). A baseline HIV-1 viral load (VL) was recorded for 5,908 patients; the median VL was 51,194 RNA copies/ml (IQR, 2,316–283,508) and 6,179/7046(88%) had initiated ART. Some 2,456 (34.9%) had a documented opportunistic infections, of whom 1,306 (18.6%) had an opportunistic fungal infection. The total number of OFI episodes was 1,632: oral candidiasis (n=1,473, 90.3%), oesophageal candidiasis (n=118; 8%), superficial mycoses (n=23; 1.6%), Pneumocystis pneumonia (PJP) (n=13; 0.8%), and cryptococcal meningitis(CM) (n=5; 0.4%). 113 (1.6%) were known to have died in the cohort.

**Conclusion:**

Approximately 1 in 5 HIV-infected patients in this retrospective cohort, most of whom had initiated ART, were clinically diagnosed with an OFI. Improved access to simple accurate diagnostic tests for CM and PJP should be prioritised for this setting.

## Introduction

The morbidity and mortality associated with HIV infection has reportedly decreased over the past decade, corresponding to increasing access to antiretroviral therapy (ART). None the less, around 1 in 3 people living with HIV (PLHIV) still present to care with advanced HIV disease. This proportion is higher in low and middle-income settings 1. Furthermore, a growing number of PLHIV are returning to care with advanced disease following a period of treatment interruption 1. Worldwide, AIDS-related death rose to 1.7 million in 2004 but has been on a steady decline to 770,000 as of 2018^2^. In Nigeria, there were 94,000 AIDS deaths in 2003 which declined to 53,000 in 2018 3. Notwithstanding this progress, the decline in AIDS-related death appears to have plateaued in recent years 1, which is mostly due to the persistent challenge of Advanced HIV Disease 4.

Globally, the proportion of people presenting with Advanced HIV Disease (AHD) has remained mostly unchanged during the past five years, although the number of people receiving ART in low- and middle-income settings has more than doubled over this period 1.

Opportunistic infections (OIs) are the leading cause of morbidity and mortality in this population with advanced HIV disease (defined as CD4+ T-lymphocyte CD4 count <200 cells/µl or World Health Organization (WHO) clinical stage 3 or 4) 5–7. An estimated 40–50% of OIS in patients with advanced HIV disease are caused by fungal pathogens 821, with an estimated 2 and 10 million annual cases of oropharyngeal and oesophageal candidiasis respectively in this population 9. Mucocutaneous and superficial fungal infections are the most common group of opportunistic fungal infections (OFIs). While these are not life-threatening, they often lead to considerable social stigma, pain, discomfort and impact on patients' quality of life.

Cryptococcus neoformans is the most common cause of meningoencephalitis among those with advanced HIV and now accounts for about 15% of AIDS-related deaths globally 10,5. While Pneumocystis jirovecii is the commonest cause of pneumonia in patients with AIDS in many parts of the world, it is dwarfed by tuberculosis in southern Africa 8,18. Histoplasma capsulatum (parts of the Americas and Africa) and Talaromyces marneffei (South and Southeast Asia) are thermally dimorphic fungi causing disseminated infections in the HIV/AIDS population 8. A recent review by Adenis et al (2018) revealed that the burden of histoplasmosis is estimated to be equivalent in incidence and even higher in deaths when compared with tuberculosis among people living with HIV in Latin America 11. Also, Coccidioides causes disease among patients with AIDS (Americas) and Emergomyces africanus has been recently described as a cause of disseminated OFI in southern Africa 8,12,19,2,5,9. These are all AIDS-defining OIs and are usually lethal unless diagnosed and treated early and appropriately^1^,^14^,^4^,^7^.

Most serious OFIs demand high-level medical skills to make a diagnosis, affect all body systems and may be misdiagnosed as other diseases (e.g tuberculosis). In addition they kill at least as many patients with AIDS comparable to tuberculosis or bacterial infections 821. There have been enormous advances in development of fungal diagnostics and antifungal agents over the past 20 years, but most of the world's population (especially in low- and middle-income countries LMICs) have not yet benefited from these advances 8. In Nigeria, the absence of diagnostic tools and antifungal agents, plus insufficient training of healthcare staff, means that the morbidity and mortality of fungal OIs remains unacceptably high.

With the increased availability of antiretroviral treatment (ART), earlier testing and treatment for HIV, the incidence of OFIs has decreased dramatically in people living with HIV in high-income countries. In contrast, in regions with a high HIV prevalence, especially sub-Saharan Africa, there is little evidence for a substantial decrease in cases of OIs 9,5. In these settings, many patients present with advanced HIV. There have been enormous advances in development of fungal diagnostics and antifungal agents over the past 20 years, but most of the world's population (especially in lowand middle-income countries LMICs) has not yet benefited from these advances 8. In countries with developed health systems, fungal OIs are usually appropriately diagnosed and treated, although many are still missed and only identified at autopsy 1,14,4,7. In Nigeria, the absence of diagnostic tools and antifungal agents, plus insufficient training of healthcare staff, means that the morbidity and mortality of fungal OIs remains unacceptably high.

There are 1,900,000 PLWHIV in Nigeria with 53,000 AIDS deaths estimated to have occurred in 2018 2; only 67% of PLHIV know their status, 53% are on treatment and 42% are virally suppressed 2. Nigeria has the 7th highest burden of tuberculosis globally 13; and an estimated 11.8% of Nigerians suffer from serious fungal infections annually and if Tinea capitis and recurrent vulvovaginal candidiasis are excluded, over 960,000 individuals are affected, with substantial mortality 14. Twenty five thousand cases of cryptococcosis are estimated to occur annually 15. Histoplasmosis is endemic in Africa, and literature reviews identified 124 documented cases in Nigeria (all before the advent of HIV)^16^. These infections account for significant morbidity and mortality in the AHD population. 1266. We aimed to determine the proportion of patients with clinically diagnosed AIDS-related OFIs at an urban ART centre in Nigeria with limited capacity for diagnosis of OFIs.

## Methods

### Study design and setting

A descriptive retrospective study was conducted over a 12-year period (April 2004-February 2016). ART-naïve, HIV-infected adolescents and adults; who were assessed for ART eligibility (including WHO HIV Stage 3/4 disease or CD4 cell count of 500 cells/ml) at the President's Emergency Plan for AIDS Relief (PEPFAR) outpatient clinic of Lagos University Teaching Hospital (LUTH), Nigeria, were included in the study. Lagos is a cosmopolitan city with an estimated population of 20 million people. This clinic has medical officers, specialist doctors, nurses, counsellors, well-staffed data office, phlebotomists etc. Patients get admitted to the hospital from this clinic. The study population included HIV-seropositive patients, 15-years-old and above. The LUTH-PEPFAR program was established in close partnership with the comprehensive HIV prevention, treatment and care services provided by Harvard University and Northwestern University.

Demographic and clinical data for each clinic visit were captured in a database. APIN (Aids Prevention Initiative in Nigeria) laboratory services this clinic. The laboratory provides for free HIV screening and confirmatory test, CD4 count, viral load, blood chemistry, hepatitis B and C testing. A panel of laboratory tests were performed at baseline and follow-up visits, including CD4 counts and HIV-1 viral load (VL). Patients/relatives pay for tests and treatment that is not covered by theEPFAR/APIN program. Infections (outside of Hepatitis B and C) diagnostics are not covered, and no fungal infection is covered. It was observed that most OIs, including OFIs, were diagnosed clinically in the studied population. Laboratory records were accessed from the PEPFAR clinic database. CD4 counts were chosen from tests closest to the period of diagnosing an OI.

### Ethical statement

The research and ethics committee of LUTH, HREC: 19/12/2008a approved this retrospective record-based study. Anonymised data only were analysed.

### Definitions (As used in Centre of study to make diagnosis)

Advanced HIV disease in this study was defined as CD4+ T-lymphocyte CD4 count <200 cells/µl or World Health Organization (WHO) clinical stage 3 or 4. The most recent CD4+ count (around the OI diagnosis) was used.

**Pneumocystis jirovecii pneumonia (PJP):** symptomatic presentation with compatible chest radiographs and clinical response to an appropriate therapeutic regimen.

**Candida oesophagitis:** symptomatic presentation of dysphagia, odynophagia or retrosternal chest pain with endoscopic evidence of lesions compatible with the diagnosis of oesophageal candidiasis or clinical response to appropriate antifungal therapy.

**Cryptococcal meningitis:** Microscopic demonstration of Cryptococcus yeasts on India ink preparation or isolation of the organism from cerebrospinal fluid sample.

**Oral candidiasis:** symptomatic presentation of white patches (plaques) in the mouth that could be wiped off leaving behind red areas that may bleed slightly.

**Superficial mycoses:** Skin, hair or nail lesions with microbiological identification using skin/ nail/ hair material or symptomatic relief with appropriate therapeutic regimens.

### Data analysis

The date of the earliest OI was compared to the date of the CD4 and RNA visits. The CD4 and RNA values were taken from the date, which was closest to the earliest OI. Patients were only considered for analysis if their visit date was within 90 days of the earliest OI date. If there was any patients with more than one value from the closest date the maximum value for CD4 and RNA was used.

Descriptive statistics were used to summarize the data by time period, age and gender. We described categorical variables using frequencies and relative frequencies. For continuous variables, an assessment of normality was carried out. If the variable was found to be normally distributed, the mean, standard deviation and range was given. Non-normally distributed variables were described using their median, range and interquartile range. We compared categorical variables using the chi square or Fisher's exact test and continuous variables using Student's t test or a non-parametric equivalent. All analyses were performed using SPSS version 22.0 (IBM SPSS Statistics. Armonk, NY: IBM Corp.).

## Results

The clinic database contained 15,098 records, each record corresponding to a clinic visit by an individual patient between the period of April 2004-February 2016. Duplicate records were removed prior to analysis. A total of 7,034 patients with available information were included in the final analysis. The majority (n=4,797, 68%) of patients were female Table 1. The mean age was 37 years (SD-10;); Table 1). The mean age at diagnosis of HIV disease for males was 41 years, (SD-9).. For females, the mean age was 35 years; SD-10. The median number of visits per patient was 2, with an interquartile range of 1 to 3. A baseline viral load was recorded for 5,908 patients; the median VL was 51,194 RNA copies/ml (IQR, 2,316–283,508). Most patients (6,179/7,034; 88%) were recorded as having initiated ART in this clinic during the study period. Of 7,034 patients, 2,456 (35%) had a documented OI. The median CD4 count from the visit that was closest to the date of the earliest OI (within 90 days) was 197 (N=49, IQR 69.5–377.5). One in five patients was diagnosed as having tuberculosis (TB) over the 12-year period (n=1,381 20%), of whom 1,282 (18%) were cases of pulmonary TB and 182 (3%) were cases of extra-pulmonary TB. A small number of the patients (17) or 0.2% had HIV encephalopathy. No confirmed cases of Toxoplasma gondii encephalitis were documented. There were only 113 (1.6%) deaths recorded in the database over the study period. See table 1.

**Table 1:** Patient demographics, CD4 counts and RNA measurements

Characteristic	Value†
Age (Years)	
Mean (Standard Deviation)	37.3 (10.0)
Minimum	8
Maximum	78
Sex	
Male	2236 (31.8)
Female	4797 (68.2)
Age at diagnosis, split by gender (Years)	
Males:	
Mean (Standard Deviation)	41.3 (9.0)
Minimum	13
Maximum	69
Females:	
Mean (Standard Deviation)	33.0 (9.7)
Minimum	8
Maximum	78
ART - initiated	6179 (88)
ART- naive	855 (12)
CD4 Count	
First Count:	
Median	184
Minimum	1
Maximum	1657
First Quartile‡	85
Last Quartile‡	328
Last Count:	
Median	418
Minimum	1
Maximum	2096
First Quartile‡	221
Last Quartile‡	612
RNA Measurement (Viral load)	
First Measurement:	
Median	51259
Minimum	0
Maximum	16,100,000
First Quartile‡	2314
Last Quartile‡	283617
Last Measurement:	
Median	200
Minimum	0
Maximum	16,100,000
First Quartile‡	25
Last Quartile‡	8597
Mortality	113 (1.6)

† Unless otherwise specified, values are number of patients (%)

‡ First quartile=25^th^ percentile; third quartile=75^th^ percentile.

### Frequency and distribution of opportunistic fungal infections

The total number of patients with an AIDS-related OFI was 1,306 (19%) see table 2. Most patients (1,197/1306) or 92%, had only one OI; 99 patients had 2 different OIs diagnosed, 6 patients had 3 different OIs and 4 patients had five OIs (this is not including multiple diagnoses of the same OI). Of the 1,306 patients, 1,284 (98%) had an episode of oral candidiasis; with 1,473 episodes documented for these 1,284 patients. One hundred and ten (8%) patients had oesophageal candidiasis with 118 episodes diagnosed in total. Thirteen (1.0%) patients were diagnosed with single episodes of PJP. Only 5 (0.4%) patients had cryptococcal meningitis diagnosed. Twenty-one (1.6%) patients were diagnosed with superficial (skin and nail) mycoses (Table 2), with 23 incidences in total. When considering multiple diagnoses of the same OFI, there were 1,632 episodes among 1,306 patients. Two hundred and forty patients had between 2 and 7 episodes of OIs, with the remaining 1,066 patients having 1 OI. The majority (90%) of the 1,632 OI episodes were Candida oral infections. Around 7% (118) of the OIs were cases of oesophageal candidiasis. PJP, superficial mycoses and cryptococcal meningitis each made up less than 1.5% of the total number of OIs reported (Table 2).

**Table 2:** AIDS-related fungal opportunistic infections

Infection	Number	Percent of total population	Percent of those with AIDS-related fungal opportunistic infections
Any AIDS-related fungal opportunistic infection	1306	18.6	-
Oral candidiasis	1284	18.3	98.3
Oesophageal candidiasis	110	1.6	8.4
Pneumocystis pneumonia	13	0.2	1.0
Cryptococcal meningitis	5	0.1	0.4
Superficial mycoses	21	0.3	1.6

There was no significant difference between the age of patients who had any OI and those who did not have an OI (38, SD 11 vs 37, SD 10; p=0.30), table 3. Male patients were significantly more likely to have an OI than female patients; 22.3% (498/2236) of males had an OI compared to 16.8% (807/4797) of females (p<0.001). See table 3.

**Table 3:** Relationship between fungal opportunistic infections and age and gender

Infection	Number	Number (%) Male	Mean age (SD), range
Any AIDS-related fungal opportunistic infection	1306	498/1305 (38.2%)	38.1 (10.7), 11–68
Oral candidiasis	1284	489/1283 (38.1%)	37.9 (10.5), 11–68
Oesophageal candidiasis	110	46 (41.8%)	39.2 (9.0), 27–60
Pneumocystis pneumonia	13	3 (23.1%)	58.5 (9.2), 52–65
Cryptococcal meningitis	5	0	-
Superficial mycoses	21	9 (42.9%)	38.6 (14.8), 13–55

PCP: Pneumocystis pneumonia

### Relationship with CD4 count

There was a statistically significant difference in the CD4 counts at both the first visit of a patient and at the last visit of a patient (both p<0.001). At their first visit, patients who had an OI during the study period had a lower CD4 count compared to those who did not have an OI during the study period (with OI: median 112; IQR 46–242 vs without OI: median 199; IQR 99–343). There appeared to be a slightly larger difference in CD4 count at a patient's last visit between those who did and those who did not have an OI during the study period (with OI: median 248; IQR 82–474 vs without OI: median 442; IQR 253–628).

### Trends of opportunistic fungal infections from 2004 to 2016

**Figure 1:**
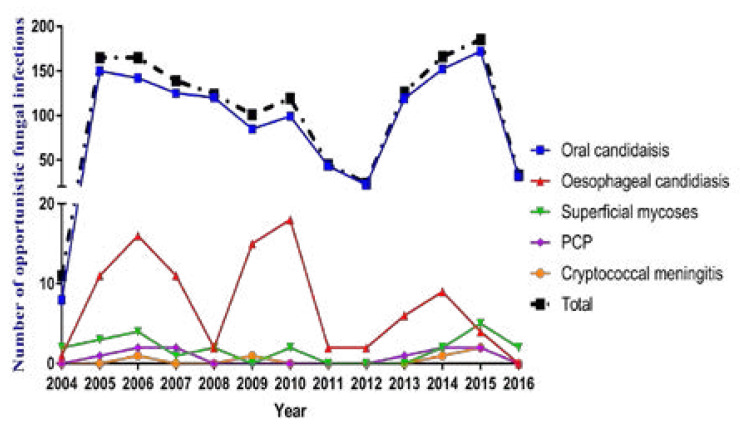
Number of clinically diagnosed opportunistic fungal infections over a 12-year period between 2004 and 2016. PCP: *Pneumocystis* pneumonia.

Among cases of OFIs with a recorded date of diagnosis (1,403 out of 1,632), most cases occurred in 2015 (185 cases) followed by 2014 (166 cases); 2005 and 2006 (164 cases each). The year 2004 had the lowest recorded cases (11) followed by 2012 (24 cases) and 2016 (33 cases). Figure 1

## Discussion

In 2013, of all persons living with HIV globally, 9% lived in Nigeria 17. OIs are associated with reversible increases in circulating viral load which could lead to accelerated HIV progression or increased transmission of HIV 5. This is contrary to the Millennium Development Goal 6; which aimed at halting and beginning to reverse the HIV epidemic by 2015. Sixty-eight percent of the studied population was female. This is in keeping with reports from other Nigerian studies, which revealed 59 – 66% 18–20. Approximately a third of the studied population in this study had an OI; higher than the 22% reported from Eastern Nigeria 19 and 26% from Central Nigeria 18 but lower than 69% reported from North Central Nigeria 20. The data from North Central Nigeria was collected prior to the HAART (highly active antiretroviral therapy) era. However our data spanned both pre-HAART and HAART eras. A recent Uganda study reported 291,168 cases among 108,619 patients over a 12-year period 21.

Approximately 1 in 5 HIV-infected patients in this retrospective study, most of whom had initiated ART, were clinically diagnosed with a fungal OI. The median CD4 count of patients was 197cells/mm^3^ and we found that there was a statistically significant difference in the CD4 counts at both the first visit and the last visit of a patient (both p<0.001). There is extensive evidence that low baseline CD4 cell count and low CD4 cell counts during ART are major risk factors for OIs 22,23. Our finding is comparable to that of a Bahraini study, which found that that CD4 count was significantly lower in HIV-infected patients with associated infections compared with those without^24^. Our study also revealed that males were significantly more likely to have an OI than females which is consistent with a South African study that found men were twice more likely than women to present with advanced HIV disease 25. A possible explanation for this might be the differences in health seeking behavior between men and women 25. Another possible explanation might be the fact that women are more likely to be diagnosed early with HIV because of the screening policy for pregnant women (the highest prevalence is in the reproductive age group) and thus women are more likely to start ART earlier. Men tend to present late to care and often with advanced HIV infection; owing to stigma, socio-cultural and religious beliefs 26, 27.

All OFIs do not have the same prognosis. While some are life-threatening; oral and oesophageal candidiasis lowers the quality of life of people living with HIV/AIDS and interfere with drug adherence and nutritional intake. Candidiasis accounted for majority of clinically diagnosed cases of fungal OIs in this study, consistent with other reports 18–20,23,24,26.

A South African post mortem study on the cause of death in HIV-infected patients within the first months of ART revealed that systemic fungal OIs occurred in 21% of cases 28. Very few systemic opportunistic fungal infections were diagnosed in the present study. Only 5 cases of cryptococcal meningitis were diagnosed over a 12-year period. This is interesting; because in the same centre, a recent study demonstrated a prevalence of 9% of cryptococcal antigenaemia 29. Also in the year 2018, three cases of cryptococcal meningitis were diagnosed, this was attributed to increased awareness by clinicians as a result of a training conducted by the Medical Mycology Society of Nigeria, and also due to availability of the diagnostic point of care kit in our laboratory. Meanwhile, a study from North Central Nigeria had reported an incidence of 36% cryptococcal meningitis in hospitalised HIV-infected patients presenting with neurological symptoms 30. Another study from the same centre on hospitalised HIV-infected patients, demonstrated that cryptococcal meningitis accounted for 8.8% of all OIs with a 12% case-fatality ratio 18.

PJP accounted for 1% of all AIDS-related mycotic infections in the present study, this is lower than 13% reported from a study in Western Nigeria 31. While diagnosis in the index study was made clinically; in the study cited above, PJP was diagnosed using a PCR assay. One of the pressing challenges in making diagnosis of PJP in our setting is the lack of awareness of the fact that simple microscopy (though not very sensitive) can be used in making a diagnosis. Another challenge is the lack of skilled personnel and infrastructure to make the diagnosis. Capacity building and awareness is urgently needed, especially given the fact that a vulnerable group (young children) is at risk.

The distribution and pattern of fungal OIs (OFIs) in the studied population did not reveal any significant change in trend over the years. In the early years of HAART (2005–2009); there appears to be a slight and steady decrease in reporting of OFIs (mainly mucocutaneous candidiasis). However, the rates subsequently picked up in 2013 with the highest incidence in 2015. We are not too sure if this is due to better reporting modalities, improved clinical diagnostic acumen, better ‘retention in care’ or virological failure. Fast, accurate diagnosis is essential to reduce deaths from AIDS 32. GAFFI's modelling suggests that making the key diagnostic tests available for only 60% of needy patients, with treatments, could save over a million lives in the next 5 years 32.

There is a dire need to improve access to essential diagnostics for these OFIs in Nigeria. Strategies will include training and retraining of healthcare workers managing these patients. Advocacy is also required to help improve access to essential diagnostics and making them affordable, especially in our setting, where patients pay for the services. Interestingly, we saw a very low (<2%) overall OFI-related mortality rate over the 12-year period. Possible explanations of this observation include poor retention to HIV care, loss to follow-up or poor documentation of death out of healthcare facilities.

## Limitations

Lack of diagnostics and skilled personnel to make appropriate diagnosis, so our findings will not reflect the true burden of disease. Most of the diagnoses were made clinically. Hospitalised HIV-infected patients in LUTH were not captured in this data. Fluidity of patient movements between different ART centres affects retention in care. However, this is one of the largest retrospective studies from Nigeria and could form the basis for further surveillance programs.

OFIs remain a significant cause of morbidity and mortality in the HIV-infected population 1. While superficial fungal infections (skin and nail) in most patients with HIV infection follow a normal pattern, atypical presentations and more severe forms are common in patients with AIDS. Diagnosis of AIDS-defining invasive OFIs such as cryptococcal meningitis has been made cheaper, easier and quicker using the CrAg LFA, which has excellent diagnostic accuracy in serum, plasma and cerebrospinal fluid 1. Despite an estimated cost of just $4 per test, no centre in Nigeria, a high HIV burden country with a PEPFAR/APIN funded ART program performs this test in routine clinical practice. PJP diagnosis has also been made faster and easier with PCR and beta D glucan assays. However these tests are neither presently accessible nor affordable in Nigeria.

## Conclusion

This study highlights the lack of diagnostics for serious fungal infections, which are a significant cause of morbidity in a high HIV burden country. There is dire need for improved access to simple diagnostic tests for AIDS-defining fungal OIs such as CM, PJP and this should be prioritised for such settings. Fast, accurate diagnosis is essential to reduce deaths from AIDS and achieve the 90-90-90 sustainable development goals by 2030.

## List of abbreviations

AIDS: Acquired immunodeficiency syndrome
ART: Antiretroviral therapy
CM: Cryptococcal meningitis
GAFFI: Global Action Fund for Fungal Infections
HIV: Human immunodeficiency virus
LMICs: Low- and middle-income countries
OFI: Opportunistic fungal infection
OI: Opportunistic infection
PJP: Pneumocystis pneumonia
PEPFAR: President's Emergency Plan for AIDS Relief
UNAIDS: Joint United Nations Programme on HIV/AIDS
WHO: World Health Organisation
